# Patient Satisfaction Toward a Tele-Retinal Screening Program in Endocrinology Clinics at a Tertiary Hospital in Riyadh, Saudi Arabia

**DOI:** 10.7759/cureus.7986

**Published:** 2020-05-06

**Authors:** Atheer Alhumud, Fadwa Al Adel, Manal Alwazae, Ghadeer Althaqib, Atheer Almutairi

**Affiliations:** 1 Ophthalmology, Princess Nourah Bint Abdulrahman University (PNU), Riyadh, SAU; 2 Surgery, Ophthalmology, College of Medicine, Princess Nourah Bint Abdulrahman University (PNU), Riyadh, SAU; 3 Ophthalmology, College of Medicine, Princess Nourah Bint Abdulrahman University (PNU), Riyadh, SAU; 4 Ophthalmology, Princess Nourah Bint Abdulrahman University (PNU), Riyadh , SAU

**Keywords:** diabetic retinopathy, tele-retinal, patient satisfaction, screening program

## Abstract

Purpose: Tele-retinal screening programs use a nonmydriatic camera for retinal imaging. These images are reviewed by ophthalmologists, for interpretation and planning of appropriate treatment and follow up. Patient satisfaction is a critical tool to assess the quality of healthcare delivery and to reframe the current screening programs. The aim of this study is to measure satisfaction toward a tele-retinal screening program among diabetics attending endocrinology clinics at a tertiary hospital in Riyadh.

Methods: This is a cross-sectional study that included a total of 163 patients recruited while attending tele-retinal screening at King Abdul-Aziz University Hospital in Riyadh, during the period between May and August 2019. A self-administrated Patient Satisfaction Questionnaire PSQ18 was used which included demographic data, diabetes history, and seven domains of satisfaction that were measured.

Results: Some 54% of the respondents were male. The average age was 44.8 years. Some 49.7% had type 2 diabetes. The mean duration of diabetes was 15.3 years. The overall satisfaction level was 80.4%. The highest satisfaction rate was in the interpersonal manner (mean 4.45) while the lowest was in accessibility to an ophthalmologist when a referral was needed (mean 3.01). Some 60% of the participants were concerned it might take a long time to be referred to an ophthalmologist when it is needed. Some 90.1% found it easier to have diabetic retinopathy (DR) screening during routine diabetes follow up. Some 23.9% did not like the idea of only seeing the ophthalmologist when it is necessary and only 9.8% had some doubts of the doctor’s ability to diagnose DR by evaluating retina photos only. No significant association was found between patient’s satisfaction and demographic background or diabetes history.

Conclusion: Patients were found to be highly satisfied with tele-retinal screening program. Mostly the reason of dissatisfaction was found in accessibility to an ophthalmologist when a referral was needed. Therefore, it is important to reassure patients that timely referral for effective intervention is performed and part of the screening policies.

## Introduction

Diabetes mellitus is a major public health problem that consumes substantial healthcare resources. In 2019, it was estimated that 463 million adults were living with diabetes worldwide [1]. In Saudi Arabia, diabetes affects nearly 3,852,000 people older than 20 years of age [1]. Diabetes-related morbidity and mortality has major physical, economical, and psychosocial implications [2-4]. Diabetes-induced complications are classified as macro and microvascular complications which can affect many organs. Diabetic retinopathy (DR) refers to progressive microvascular changes that affect retinal circulation as a result of chronic hyperglycemia. By 2030, the number of people with DR are expected to reach 191 million worldwide [5]. It is one of the major causes of visual impairment among Saudi adults [6]. Initially, DR is asymptomatic, therefore regular screening, early diagnosis, and timely treatment improve clinical outcomes [7-8]. People with type 1 diabetes should start DR screening after five years of the onset of the disease, while those with type 2 diabetes should start screening once they are diagnosed. Unfortunately, compliance to regular screening among Saudi diabetic patients is still unsatisfactory [9].

Multiple approaches like telemedicine services have been developed to achieve higher compliance rates with regular screening [10]. The use of telemedicine technology as a screening tool for DR has evolved from a research tool to a clinical tool [11]. Tele-retinal screening have been reported to be an effective method to diagnose patients with sight-threatening conditions [12]. Digital retinal imaging has been used and it significantly improves screening rates over the conventional screening methods [13]. In our tele-retinal screening program a specialized nonmydriatic camera for retinal imaging is used in nonophthalmic diabetes clinics; these images are sent to be reviewed by ophthalmologists who provide their interpretation and decide if the patient need further detailed assessment or management.

Patient satisfaction on the provided care is considered as an important measure of the quality of healthcare [14]. It has been reported that patient satisfaction is correlated with several positive outcomes including higher compliance rate with medical advises and follow up screening visits [15]. Satisfaction feedback from patients regarding any screening program can provide valuable data to help shape and improve the framework of current and future screening programs [16]. There are a few published studies that evaluated patient's satisfaction level regarding tele-retinal screening program, none of which were done in Saudi Arabia. Therefore, the aim of this study is to determine Saudi patient's satisfaction toward the tele-retinal screening program in the endocrinology clinics at King Abdul Aziz University Hospital Diabetes Center in Riyadh, Saudi Arabia.

## Materials and methods

Study design 

This was a cross-sectional questionnaire-based study conducted in the endocrinology clinics at King Abdul-Aziz University Hospital in Riyadh, Saudi Arabia during the period between May 2019 and August 2019.

Sample size calculation 

A convenience sample was used to recruit diabetic patient. A previous literature showed that 88% of patients were satisfied with tele-retinal screening program [17]. Using G-power program for calculating the minimal sample size with 95% level of significance (the minimal β=0.2), and a power of 80% (α=0.05) the minimal sample size needed was 163 and it was increased to 180 to compensate for incomplete data.

Patient recruitment

Participants were diabetic patients who have had their baseline ophthalmological examination done previously. They were recruited while attending their diabetes follow up and retinal imaging appointment. Patients with both type 1 and type 2 diabetes were included. Participants who were aged below 18 were excluded from the study. 

Material 

Data were collected by self-administered questionnaire. A modified version of The Patient Satisfaction Questionnaire (PSQ18) which was developed by Grant N. Marshall and Ron D. Hays was used to collect the data. The approval to use the questionnaire was obtained from the authors.

The questionnaire is divided into three main sections:

1- Sociodemographic data: including age, sex, marital status, level of education, employment status, and residency area.

2- Diabetic history: including duration, type of diabetes, type of medications, last HbA1c, last retinal imaging, any history of diabetic-related eye disease, and any history of intravitreal injections or laser treatment.

3- Satisfaction level: contains 16 closed ended questions with five points answers on the Likert scale evaluating seven aspects of satisfaction: General satisfaction, Technical quality, Interpersonal manner, Communication, Time spent during screening, Accessibility to tele-retinal screening, and Accessibility to an ophthalmologist when a referral is needed. The financial aspect of satisfaction was not included in the questionnaire as tele-retinal screening is delivered freely in the hospital. 

The questionnaire was translated to Arabic forward and backward by two independent translators and pretested on 20 participants to assess the feasibility and the clarity of the asked questions. 

Validity and reliability

Content validity and face validity of the modified version were evaluated by three independent experts, an ophthalmologist, and two experts in research methodology. Internal consistency of the questionnaire was evaluated, Cronbach's alpha coefficient was calculated and had a good result of 0.76.

Data analysis

Data were analyzed using Statistical Package for Social Science Program (SPSS) VERSION 21. Descriptive statistics in terms of mean, standard deviation, median, range, and percentage were used to describe characteristics of the studied sample. Means of different variables were compared using t-test and one-way analysis of variance (ANOVA). Regression analysis was used to evaluate the effect of independent variables (demographics and diabetes history) on the overall satisfaction. A p-value less than 0.05 was considered as a statistically significant result.

Scoring system

Each question had five options (strongly agree; agree; neutral; disagree; strongly disagree) each option represent a score ranging from one to five, where five represents the highest satisfaction and 1 for the poorest satisfaction. The mean score of each item of satisfaction was calculated and compared with other aspects of satisfaction.

Ethical consideration 

The study was approved by King Saud University Institutional Review Board. The participants were informed about the study and consented verbally before enrolment. Confidentiality was assured with regard to patient's identifiable information and the participation was voluntary. 

## Results

Demographic data

In total, 163 patients completed the questionnaire. Some 75 of the participants were female and 88 were male. The age ranged from 18 to 82 years, with a mean of 44.8 years. Some 63.2% were married, 26.4% single, 3.1% widowed, and 7.4% were divorced. Regarding the educational level, 3.1% had no education at all, 1.2% had elementary school education, 7.4% and 22.1% had middle and high school education, respectively. The majority 66.3% of the participants had college or higher level of education. Some 38% were employed and 62% were unemployed including students and retired. Some 81% were living in the same city Riyadh and only 19% were from outside the region. The participants’ demographic background distribution is shown in Table 1.

**Table 1:** Participants' demographic distribution (n=163).

Variables	n (%)
Gender	
Male	88(54.0)
Female	75(46.0)
Age	
18-37 years	58(35.6)
38-57 years	60(36.8)
58-77 years	43(26.4)
78-97 years	2(1.2)
Marital status	
Single	43(26.4)
Married	103(63.2)
Widowed	5(3.0)
Divorced	12(7.4)
Educational level	
None	5(3.1)
Elementary school	2(1.1)
Middle school	12(7.4)
High school	36(22.1)
College or higher	108(66.3)
Employment	
Nonemployee	101(62.0)
Employee	62(38.0)
Residency	
Riyadh	132(81.0)
Outside Riyadh	31(19.0)

Diabetes status

The majority 49.7% of participants had type 2 diabetes, while 42.3% had type 1. Surprisingly, 8.0% did not know which type of diabetes they had. The mean duration of diabetes was 15.3 years. The mean HbA1c level was 8.3 mmol/L. The majority of patients 36.8% were on insulin for the control of their diabetes. While 31.9% were using both oral hypoglycemic agents and insulin. Some 30.7% were using oral medications only. None of the patients were on diet alone. Some 0.6% did not use any treatment modality for their diabetes. 

Some 81.6% of participants had their last retinal imaging within one year. Regarding diabetic induced ocular damage 25.1% of participants self-reported that diabetes has caused some damage to their eyes, while 35% did not know if their eyes were affected by diabetes. The majority of patients never had an eye injection or a laser treatment for DR 93.3% and 86.5%, respectively. Table 2 represents the distribution of participants according to their diabetes history.

**Table 2:** Participants' diabetes history (n=163). DM, diabetes mellitus

Variables	n (%)
Type of DM	
Type 1	69(42.3)
Type 2	81(49.7)
I do not know	13(8.0)
Duration	
Less than 10 years	45(27.6)
11 to 20 years	90(55.2)
21 to 30 years	24(14.7)
More than 30	4(2.5)
HbA1c	
Less than 7 mmol/L	34(20.9)
More than 7 mmol/L	129(79.1)
Diabetes control	
None	1(0.6)
Oral hypoglycemic	50(30.7)
Injection	60(36.8)
Both oral hypoglycemic and injection	52(31.9)
Last retinal image	
One year or less	133(81.6)
More than one year	30(18.4)
Diabetes-induced ocular problem	
Yes	41(25.1)
No	65(39.9)
I do not know	57(35)
History of ocular injection	
Yes	7(4.2)
No	152(93.3)
I do not know	4(2.5)
History of ocular laser	
Yes	18(11.0)
No	141(86.5)
I do not know	4(2.5)

Patient satisfaction

Table 3 shows the mean satisfaction score of different domains of satisfaction. The highest satisfaction rate was observed in the interpersonal manner 4.45 ± 0.65, followed by general satisfaction 4.28 ± 0.68, time spent during screening 4.28 ± 0.68, communication 4.09 ± 0.75, accessibility to tele-retinal screening 4.05 ± 0.77, and technical quality 4.02 ± 0.65. The least satisfaction rate was in accessibility to ophthalmologist when referral is needed 3.01 ± 0.89. The overall satisfaction was 80.4%.

**Table 3:** Mean value of different aspects of satisfaction. SD, standard deviation

Domain of satisfaction	Mean ± SD
General satisfaction	4.28 ± 0.68
Technical quality	4.02 ±0.65
Interpersonal manner	4.45 ± 0.65
Communication	4.09 ± 0.75
Time spent during screening	4.28 ± 0.68
Accessibility to tele-retinal screening	4.05 ± 0.77
Accessibility to ophthalmologist when referral is needed	3.01 ± 0.89

Some 94.5% of patients agreed that they are totally happy with the use of tele-retinal imaging for their diabetic screening. Some 94.4% agreed that photographers who took their retinal image were kind during the procedure. Some 90.1% of participants found it easier to have their retinal screening during their diabetes follow up rather than having a separate screening appointment with an ophthalmologist. Some 68.8% of participants did receive information about the benefits of the tele-retinal screening program. Only 9.8% of respondents had doubts about the doctors’ ability to diagnose DR based on the interpretation of retinal images only. 

 

Some 60% of participants thought that a referral to an ophthalmologist, when needed, will take a long time. This was found to be the most reported reason for dissatisfaction. Some 24.4% were not sure if the machine was able to fully capture any retinal pathology. Some 23.9% of patients did not like the idea of only seeing the eye doctor when it is necessary (i.e., if further assessment or treatment was needed). Table 4 shows the percentage of most reported items of dissatisfactions.

**Table 4:** Most reported items of dissatisfactions.

Items of dissatisfaction	Agree	Disagree
It might take long time to be referred to eye doctor when needed	60.0%	40.0%
I am not sure if this screening technique could detect my eye disease	24.4%	75.6%
I do not like the idea of only seeing the eye doctor when it is necessary	23.9%	76.1%

Comparison of satisfaction according to socio-demographic data and diabetes-related history

The mean score of satisfaction for the age group between 38 and 57 years was significantly higher (4.15 SD 0.40) than for the age group between 58 and 77 years (3.88 SD 0.45) p=0.019.

No statistically significant differences were found in the satisfaction scores of patients by gender, educational level, employment status, or residency.

The mean score of satisfaction for patients had their last retinal imaging with one year or less was higher than that for patients who had it more than one year (4.07 SD 0.46, 3.80 SD 0.48, respectively), this was found to be statistically significant (p=0.005). 

No other statistical differences in the mean satisfaction scores according to other diabetes-related history. Table 5 shows the mean satisfaction according to socio-demographic data and diabetes-related history.

**Table 5:** Mean score of satisfaction according to socio-demographic data and diabetes-related history. SD, standard deviation

Variables	Mean ± SD	p
Gender		
Male	4.01 ± 050	0.714
Female	4.03 ± 0.44	
Age		
18-37	4.01 ± 0.52	0.006
38-57	4.15 ± 0.40	
58-77	3.88 ± 0.45	
78-97	3.37 ± 0.70	
Marital status		
Single	4.00 ± 0.51	0.076
Married	4.06 ± 0.47	
Widowed	3.50 ± 0.25	
Divorced	3.98 ± 0.34	
Educational level		
None	4.00 ± 0.34	0.868
Elementary	4.03 ± 0.57	
Middle school	3.90 ± 0.46	
High school	3.98 ± 0.47	
College or higher	4.04 ± 0.49	
Employment status		
Non-employee	3.99 ± 0.45	0.358
Employee	4.06 ± 0.51	
Residency		
Riyadh	4.03 ± 0.48	0.369
Outside Riyadh	3.95 ± 0.43	
Type of diabetes		
Type 1	4.08 ± 0.49	0.361
Type 2	3.97 ± 0.48	
I do not know	3.98 ± 0.33	
Duration of diabetes		
10 years or less	4.17 ± 0.42	0.058
11-20 years	3.94 ± 0.49	
21-30 years	4.06 ± 0.44	
31 or more	3.87 ± 0.53	
HbA1C		
Less than 7 mmol/L	4.16 ± 0.45	0.051
More than 7 mmol/L	3.98 ± 0.47	
Control of diabetes		
None	4.31 ± 0.00	0.912
Oral hypoglycemics	4.04 ± 0.48	
Injections	4.01 ± 0.49	
Both	4.00 ± 0.45	
Last retinal image		
One year or less	4.07 ± 0.46	0.005
More than one year	3.80 ± 0.48	
Diabetes-induced ocular problem		
Yes	3.96 ± 0.49	0.264
No	4.09 ± 0.50	
I do not know	3.98 ± 0.42	
History of ocular injection		
Yes	4.14 ± 0.26	0.390
No	4.02 ± 0.40	
I do not know	3.73 ± 0.35	
History of ocular laser		
Yes	3.90 ± 0.41	0.230
No	4.04 ± 0.48	
I do not know	3.73 ± 0.35	

Regression analysis

Regression analysis was carried out to examine the correlation between the overall satisfaction score as a dependent variable and each of the demographic background information and diabetes history as an independent variable. No correlation was observed between any of the participant’s characteristics and the overall satisfaction score.

## Discussion

Diabetic retinopathy is one of the major targeted diseases by telemedicine studies, as it is considered to be the most common cause of irreversible blindness due to low compliance rate of annual screening [18-19]. In a systematic review performed on the application of teleophthalmology services in Europe, patients’ satisfaction was found to be an important factor which plays a role in the effective implementation of teleophthalmology [18]. Patients’ satisfaction towards tele-retinal screening was assessed in multiple studies where they use a questionnaire which collects information about different aspect of satisfaction including waiting time, convenience, and the quality of the services [18]. In a study carried out by Kurji et al. 88% of the participants preferred a teleophthalmology-based screening over a traditional ophthalmologist-based screening [17]. Another study done by Rani and colleagues found that 99% of their patients were satisfied with teleophthalmology [20]. A qualitative Australian study concluded that patients were highly satisfied with remote DR screening [21].

In our study, the overall satisfaction was 80.4%, which is lower than all of the above-mentioned studies. This could be due to the fact that 81% of our participants were from the same city where the tele-retinal screening program was implemented; as opposed to the other studies that had their screening programs implemented in rural areas which helped patients in terms of travel cost and accessibility. Kurji et al., Rani et al., and multiple other studies in the literature found that convenience is the primary reason of satisfaction with teleophthalmology screening [17, 20]. One study done by Valikodath et al. reported that patients who had better access to healthcare did not consider tele-retinal DR screening as a convenient tool, but this did not influence their willingness to use it [22].

The main goal of implementing tele-retinal screening program is to improve accessibility and regular screening rate among diabetic patients. A 16.3% improvement of DR annual screening rate (from 40.6% to 56.9%) was achieved after initiating a tele-retinal DR screening program in primary care clinics in Los Angeles, United States [23]. Silva et al. reported increase in the rate of annual retinal examination from 50% to 75% [24]. A recent study done in Riyadh, Saudi Arabia in 2019 showed a suboptimal compliance (61.4%) to regular attendance of DR screening among Saudi adults [25]. Another study conducted in Riyadh revealed that only 45% of the patients had DR screening done within one year [26]. In our study we had a much higher compliance as 81.6% of our participants had their last retinal imaging done within one year. If this higher percentage is only due to the implementation of a tele-retinal screening program, then an improvement of 20.2%-36.6% is found respectively when compared to the previously mentioned two studies done in Riyadh.

Beside accessibility to tele-retinal screening, communication and co-ordination of care is considered as a vital component when implementing an effective tele-retinal screening service [21]. The lowest rate of satisfaction in our study was found to be in accessibility to an ophthalmologist when a referral was needed as 60% of the respondents were concerned that it might take a long time for their referral. One qualitative Australian study found that one of the challenges they faced was providing timely referral for patients with positive results as many of those patients did not receive any ophthalmology follow up after screening [21]. They attributed this issue to the large number of health providers involved in the screening program and the lack of formal communication between healthcare providers. The study suggested using electronic clinical notes for better communication and to ensure providing follow up appointments for patients with positive results [21]. In our tele-retinal screening program, we use electronic files to access the patient’s information and document the results of their tele-retinal screening visits, as well as schedule follow up appointments for patients who need further evaluation and treatment. This is important to ensure an effective implantation of the screening program.

Trust is a crucial component when providing healthcare [27]. In a meta-analysis performed on the effect of trust on the clinical outcomes, patients who trust their healthcare professionals were found to be more satisfied with the provided treatment [27]. In our study, 50.3% of respondents trust the machines’ ability to capture their retinal pathology. Some 71.1% of our patients believe in the doctors’ ability to diagnose DR based on the interpretation of retinal images alone. George et al. study, showed a higher level of confidence on the ability of machine and doctors to capture and diagnose their retinal disease, 66.4% and 86.8%, respectively [28]. In their study, they believed that the shortage of healthcare delivery in South rural India compared to other populations explains the higher level of satisfaction with tele-retinal screening [28].

Multiple studies in the literature examined the relationship between patient satisfaction and demographic background including age, gender, level of education and health status and their findings are inconsistent [29-30]. In our current study there was no correlation between the level of satisfaction and patients' demographics or diabetes-related history. Similarly, a study done in rural India, reported that patients’ demographics are not predictors of patient satisfaction with teleophthalmology [28]. However, Valikodath et al. found that patient attitudes toward telemedicine is influenced by their health status, and not by demographic characteristics [22]. They found that patients with long history of diabetes prefer more personal method of screening rather than machine-based model as they value the relationship with their physician [22].

## Conclusions

Tele-retinal screening program appears to be well accepted by the patients in Riyadh region. The main reason for patients’ dissatisfaction was their assumption that a referral to an ophthalmologist, when indicated will take a long time. Therefore, it is important to reassure patients that timely referral for effective intervention is performed and part of the screening policies. Further studies investigating care providers’ attitude to teleophthalmology services, the impact of this screening model on adherence to regular screening, and its clinical outcomes are needed.
